# Neural Coding of Multiple Linguistic Components During Reading Comprehension Across Development

**DOI:** 10.1162/NOL.a.286

**Published:** 2026-09-14

**Authors:** Jie Chen, Xiaoxia Feng, Jia Zhang, Chan Tang, Linyan Liu, Siyi Fan, Ningxin Zhao, Yang Lei, Xiujie Yang, Xiangzhi Meng, Guosheng Ding

**Affiliations:** State Key Laboratory of Cognitive Neuroscience and Learning & IDG, McGovern Institute for Brain Research, Beijing Normal University, Beijing, China; Beijing Key Laboratory of Applied Experimental Psychology, National Demonstration Center for Experimental Psychology Education, Faculty of Psychology, Beijing Normal University, Beijing, China; School of Psychological and Cognitive Sciences, Beijing Language and Culture University, Beijing, China; Mental Health Education Center, Zhengzhou University, Zhengzhou, China; School of Psychological and Cognitive Sciences, Beijing Key Laboratory of Behavioral and Mental Health, Peking University, Beijing, China; PekingU-PolyU Center for Child Development and Learning, Peking University, Beijing, China

**Keywords:** combination coding, language network, neural representations, semantic, situation model, syntactic

## Abstract

Understanding how multiple linguistic components are represented within the human language network, and how these representations change across development, remains an important open question. This study examines whether the language network integrates linguistic components through regions dedicated to single components (single-component coding) or through regions representing combined components (combination-based coding), and how these representational patterns differ across development. We recruited 64 participants (34 children and 30 adults) to complete a hierarchical reading task involving pseudowords, words, sentences, and stories, enabling assessment of neural substrates for individual language components (semantics, syntax, situation model) and their combinations. Based on this task structure, we constructed seven cognitive models targeting individual components and their combinations. Model-based multivariate regression revealed overlapping engagement of left frontal and temporal regions across all models in both groups, indicating multifunctional regions supporting combination-based coding. Compared to children, adults showed more spatially focal model correspondence within core language regions. Moreover, clustering based on model-fitting performance identified three functional clusters with marked developmental differences, broadly corresponding to right frontotemporal regions (Cluster 1), left anterior frontotemporal regions (Cluster 2), and left posterior frontotemporal regions (Cluster 3). Further statistical analyses showed that adults exhibited stronger Combination Code representations in Cluster 2, whereas children relied more on Single Code representations in Cluster 3, suggesting a developmental shift toward integrated coding in anterior networks. Together, these findings reveal that the language network supports combination-based coding through functionally organized clusters, and that the representational roles of anterior and posterior regions undergo reorganization from childhood to adulthood.

## INTRODUCTION

Human language, a cornerstone of human cognition, is supported by a well-organized neural network primarily located in the temporal and frontal lobes, demonstrating left-hemisphere dominance (Fedorenko et al., 2010; Pallier et al., 2011). This fronto-temporal network exhibits robust and selective responses to linguistic stimuli, with consistent activation patterns across modalities and languages (Fedorenko et al., 2010; Regev et al., 2013; Scott et al., 2017). Language comprehension involves complex hierarchical cognitive processing, including semantic interpretation, syntactic parsing, and situational modeling. While previous studies emphasized delineated cortical areas for distinct linguistic functions, converging evidence indicates that certain regions within the language network are consistently involved in multiple linguistic processes, underscoring the functional multiplicity of its regions for flexible information integration (Hodgson et al., 2021; Shain et al., 2024; Vigneau et al., 2006). However, how the brain integrates multiple linguistic components to support efficient comprehension, and how the functional organization of the language network underlying such integration evolves from childhood to adulthood, remain poorly understood.

Language comprehension unfolds hierarchically, progressing from semantic and syntactic computations to the construction of a situation-level model—a process in which semantic representations provide the foundation for syntactic organization, which in turn supports the interpretation of broader contextual and event-level information (Kintsch, 1988; van Dijk & Kintsch, 1983; Zwaan & Radvansky, 1998). While the integration of linguistic components is fundamental to language comprehension, how the brain accomplishes this remains debated, as contradictory evidence exists. One view posits that the language network operates through a single-component coding mechanism, which links specific brain regions to distinct linguistic processes such as semantic, syntactic, and situational processing, with integration emerging from their coordinated interactions. Supporting evidence comes from findings that subregions of the left inferior frontal gyrus are differentially sensitive to semantic versus syntactic information (Dapretto & Bookheimer, 1999; Goucha & Friederici, 2015; Vigneau et al., 2006). According to this view, failures in single-component processing may disrupt overall language comprehension (Dick et al., 2001). Alternatively, there may be a combination-based coding mechanism in the language network, whereby a subregion encodes and integrates two or more language subcomponents. Although this mechanism may introduce redundancy in neural resources, it can enhance processing robustness and maintain stable comprehension as linguistic input unfolds continuously (Brodbeck et al., 2022; Mollica et al., 2020). Supporting evidence includes extensive overlapping activation across linguistic components in the temporal lobe and other fronto-temporal regions (Hodgson et al., 2021; Shain et al., 2024; Visser et al., 2010; Wang et al., 2023). A pivotal question, therefore, is to investigate whether the language network employs single or combination-based coding mechanisms (strict one-to-one functional mapping vs. overlapping, interactive engagement) to process the multiple components of language. Besides, if combinatorial mechanisms are involved, it is essential to characterize the nature of the involved hierarchical information integration.

Furthermore, recent evidence suggests that the language network may operate via a distributed functional architecture, in which spatially distant brain regions can subserve the same cognitive function, unconstrained by their underlying structural anatomy. For instance, neural responses to semantic content (Deniz et al., 2019; Huth et al., 2016; Zhang et al., 2020) and syntactic complexity (Blank et al., 2016) are widely distributed across frontal and temporal language areas, but not confined to specific regions. Further supporting this view, recent intracranial recordings have identified spatially segregated yet functionally homogeneous clusters that respond selectively to distinct linguistic units (Regev et al., 2024; Woolnough et al., 2023). Although these intracranial studies have delineated functional clustering based on temporal dynamics, the spatial organization that supports the processing of multiple language components remains largely unexplored. In other words, the internal architecture of the language network supporting the integration of different linguistic elements is still unknown.

Additionally, although adults’ brain could efficiently integrate various linguistic information for rapid comprehension, it remains unclear how this efficient neural pattern is formed. Studies found that a foundational language network is already established by approximately 4 years of age (Ozernov-Palchik et al., 2025). By late childhood (ages 9–10), the left inferior frontal gyrus pars opercularis (LIFGoper) shows selective sensitivity to syntactic information, while temporal regions such as the superior temporal gyrus and the middle temporal gyrus (MTG) respond to both syntactic and semantic cues (Wang et al., 2024). These findings suggest that while the basic language network is established early, the complex combination-based coding mechanism within it may develop gradually. Therefore, comparing children with adults is essential to understanding how this network matures to support efficient and stable linguistic integration. One hypothesis to be examined is that with greater experience, specific brain regions develop the ability to integrate multiple cognitive components. This allows them to acquire more complex functional profiles while reducing the reliance on widespread regional activation, ultimately forming a more focal and efficient cortical network.

The current study aims to investigate the internal functional organization of the language network by examining how it processes distinct linguistic components and how this organization develops from childhood into adulthood. To address these questions, we implemented a hierarchical reading task encompassing conditions of pseudowords, words, sentences, and stories. Within this hierarchical task framework, differential weighting of these conditions allows us to simultaneously model neural responses to different linguistic units and to disentangle brain activity associated with semantic, syntactic, and situation model components, as well as their various combinations. Accordingly, we constructed seven cognitive models: three single-code models, each using a binary weighting to individually isolate semantic, syntactic, or situational model components; and four combination-code models, which represent the integration of two or three components (e.g., semantic + syntactic, or semantic + syntactic + situational), respectively, by ternary or quaternary encoding schemes.

We first conducted model-based multivariate regression analyses for adults to identify general activation patterns associated with individual linguistic components and their combinations. Besides, we also conducted the above analyses on children and further quantified the differences between children and adults in brain activation to language components. If the brain displays selective and spatially segregated responses to single-code models, it suggests that language processing adheres to a single-coding principle. Conversely, activity to combination-code models, along with observed spatial overlaps among regions associated with distinct single-code models would signify a mechanism rooted in combination-based coding. Beyond activation analyses, we performed representational similarity analysis (RSA) within the language network and evaluated the model fit for each language-related region of interest (ROI). Based on the fitting profiles across twelve ROIs and seven cognitive models, we applied data-driven hierarchical clustering to reveal the internal organizational principles of these language regions, identifying distinct functional clusters. Finally, we quantified each model’s contribution to the cluster patterns and examined developmental differences (adults vs. children) in these contributions for models that showed significant predictive power in either group.

Addressing our research questions, we hypothesized that while both adults and children rely on a combination-based mechanism for language processing (overlapped across single and combination models), the underlying neural substrates differ. In adults, multifunctional regions that support combination-based coding are more focal, corresponding to a more efficient pattern in which fewer brain regions process more components. In children, activation is more widespread across models, likely due to less mature integration abilities, necessitating the recruitment of a broader neural network. Furthermore, we propose that the language network supporting this combination-based coding is organized into functionally homogeneous clusters. Compared to children, adults’ clusters integrate different and more linguistic components, implying a maturational shift toward a more highly integrated organizational architecture from childhood to adulthood.

## METHODS AND MATERIALS

### Participants

A total of 64 participants were recruited for the current study: 34 children (mean age = 11.82 years, *SD* = 1.13, 23 males) from grades 4–6 at primary schools in Beijing, and 30 adults (mean age = 23.50 years, *SD* = 2.40, 12 males) who were local college students. In the fMRI scanning, each participant performed a hierarchical reading task that required passive reading of linguistic stimuli ranging from pseudowords and words to sentences and stories. All participants were native speakers of Mandarin Chinese, right-handed, and had normal or corrected-to-normal vision. The study received ethics approval from Beijing Normal University, and written informed consent was obtained from all adult participants and from the parents or legal guardians of all child participants. None of the participants reported having a history of neurological diseases or psychiatric disorders.

### Behavioral Measures

Three standardized assessments were administered to characterize the linguistic and reading profiles of both groups.

#### Rapid automatized naming

Adapted from Denckla and Rudel (1976), participants were presented with a number matrix arranged in seven rows and five columns and were asked to read the numbers from left to right and top to bottom as quickly as possible. The task was completed twice, and the score reflects the average time (in seconds) taken to complete one reading of the number matrix, with lower scores indicating faster and more automatic naming.

#### Character reading fluency

Adapted from the HKT-SpLD subtest (Pan & Lin, 2024), this task assessed character-level reading fluency. Participants read 200 characters aloud as quickly as possible within 1 min, with the total number of correctly read characters serving as the score.

#### Verbal fluency

Following Lezak et al. (2004), this semantic fluency task required participants to generate as many words as possible within 1 min belonging to each of four semantic categories (animals, fruits, work-related words, and words containing the sound /fa/). The final score was the average number of correct responses across the four categories.

### Hierarchical Reading Task Design

The hierarchical reading task comprised four block-based conditions corresponding to distinct language components across different levels (pseudowords, words, sentences, and stories), enabling the investigation of the neural mechanisms underlying individual components as well as their hierarchical combinations. In the pseudoword condition, participants saw lists of pseudowords composed of orthographically and phonetically legal but meaningless Chinese characters; in the word condition, they saw lists of real words; in the sentence condition, they saw individual sentences that lacked semantic connections with each other; and in the story condition, they saw consecutive sentences that together formed a coherent narrative.

**Figure 1.**
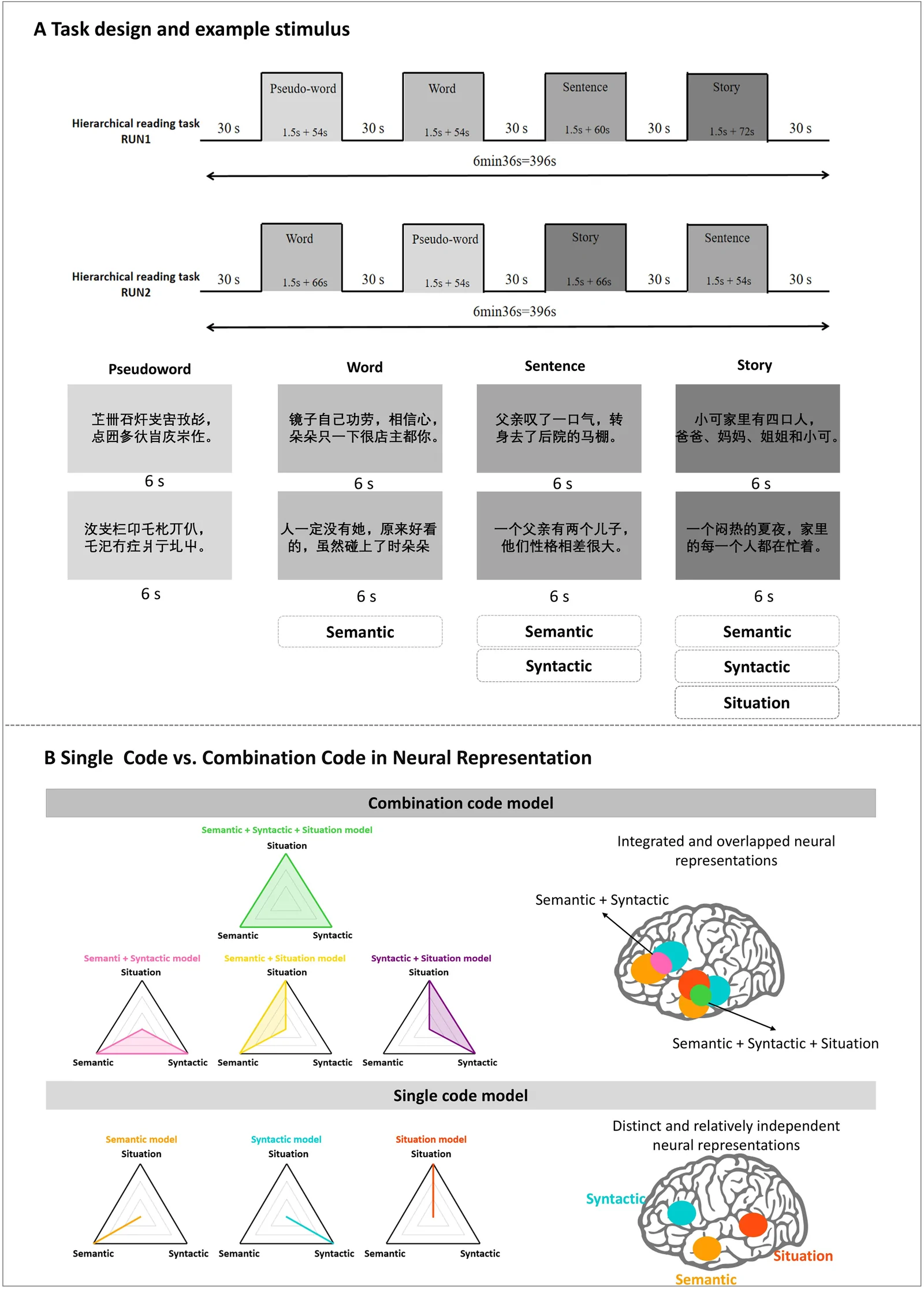
Experimental design and theoretical framework of the hierarchical reading task. (A) The two runs of the hierarchical reading task (run1 and run2) were designed such that each run comprised blocks of four conditions (pseudowords, words, sentences, stories), each lasting approximately 1 min, with 30-s fixation periods presented between blocks. The presentation order of the blocks was pseudo-randomized across runs. Example stimuli were provided for each condition. (B) The seven cognitive models are defined by combinations of three language subcomponents (semantic, syntactic, and situational), illustrating single versus combination code models of neural representation. Under a single-code mechanism, neural responses to individual language components are spatially segregated; under a combination-code mechanism, regions associated with distinct single-code models show spatial overlap.

The task included two runs, resulting in a total scanning time of 13 min and 12 s per participant. Each run lasted 6 min and 36 s and comprised one block per condition, yielding a total of 4 blocks. In each trial, stimuli were presented in the form of sentences, which contained six to fifteen pseudowords/words on each screen. A block comprised 9–12 trials and lasted approximately 1 minute (Aboud et al., 2019; Wang et al., 2015; Zhou et al., 2021). Each block was followed by a 30-s rest period, preceded by a 1.5-s cue to prepare for the next block. The example and materials for different conditions were provided in Figure 1A.

This task combined a passive viewing setting with a hierarchical reading structure, creating a child-friendly paradigm that controlled for variations in cognitive performance during the fMRI task, thereby enabling direct comparisons of brain activities between children and adults. Three measures were taken to promote and assess participant engagement. First, prior to scanning, all participants completed a practice session using materials that were distinct from those used in the formal in-scanner experiment, ensuring that they were familiar with the task requirements before entering the scanner. Participants were instructed to read carefully, following the conventional left-to-right and top-to-bottom reading order. Second, during the scanning session, eye movements were monitored in real time using an eye-tracking system to verify that participants actively fixated on the screen and attended to the presented stimuli throughout the task, thereby providing online evidence of visual engagement with the reading materials. Third, following the scanning session, all participants underwent a post-scan verbal comprehension check, in which they were asked to recall the content of the stories. All participants were able to recall the main content of the stories, indicating that they had engaged in the intended linguistic processing during the MRI scan.

### fMRI Data Acquisition and Preprocessing

All MRI data were obtained on a SIEMENS PRISMA 3-Tesla scanner at Peking University. All volumes were acquired using a multiband echo-planar imaging (MB-EPI) sequence with the following settings: TR = 750 ms, TE = 30 ms, flip angle = 65°, FOV = 200 × 200 mm^2^, slice number = 55, multiband acceleration factor = 5, slice thickness = 2.5 mm, voxel size = 2.5 × 2.5 × 2.5 mm^3^, scanning method is interleaved scanning. For structural images, the specific parameters are: TR = 2,530 ms, TE = 3.1 ms, flip angle = 7 °, FOV = 205 × 205 mm^2^, thickness = 0.8 mm, voxel size of 0.8 × 0.8 × 0.8 mm^3^. Preprocessing of fMRI data was performed using DPASFA (Yan et al., 2016). The steps included: (1) Removal of the first 10 time points from the functional images, followed by slice timing correction and realignment; (2) outlier volumes caused by head motion were repaired using the Artifact Repair toolbox (Li et al., 2022; Mazaika et al., 2007); (3) co-registration of functional and T1 images; (4) segmentation of T1 images into gray matter, white matter, and cerebrospinal fluid; (5) normalization of functional images to the T1 image using unified segmentation, followed by removal of low-frequency drift, and regression of gray matter, white matter, cerebrospinal fluid, and head motion using the Friston 24-parameter model; and (6) linear trend removal and temporal filtering with a bandpass filter (a cutoff of 1/128 Hz).

### Univariate Activation Analyses at the Whole-Brain Level

We first present activation maps for each condition contrasted with the pseudoword baseline (word > pseudoword, sentence > pseudoword, story > pseudoword) to establish the activation patterns of language-related components. Subsequently, to characterize activation patterns specific to individual language subcomponents, we conducted pairwise contrasts between conditions to isolate syntactic processing (sentence > word) and situation model processing (story > sentence) beyond the lexical-semantic processing captured by the word > pseudoword contrast (Supplementary Figures S1–S2; Supporting Information can be found at https://doi.org/10.1162/NOL.a.286).

### Model-Based Multivariate Regression Analyses at the Whole-Brain Level

Contrast-based analyses, however, can only detect activation differences between two specific conditions, limiting their ability to capture a region’s overall response profile across several conditions. We therefore conducted model-based multivariate regression analyses at the whole-brain level, using seven models collectively referred to as the Single Code models and Combination Code models, which represented the cognitive components of semantic, syntactic, and situational processing and their pairwise or full combinations. Specifically, Single Code models differentiate among any of the three cognitive components by assigning weights to different linguistic units: non-semantic versus semantic [pseudowords vs. (words, sentences, and stories) = 1, 2, 2, 2], non-syntactic versus syntactic [(pseudowords, words) vs. (sentences, and stories) = 1, 1, 2, 2], and non-situational versus situational [(pseudowords, words, sentences) vs. stories) = 1, 1, 1, 2]. In addition, three Combination Code models, each represents a combination of two Single Code models: semantic–syntactic [pseudowords vs. words vs. (sentences and stories) = 1, 2, 3, 3], semantic–situational [pseudowords vs. (words and sentences) vs. stories = 1, 2, 2, 3], and syntactic–situational [(pseudowords and words) vs. sentences vs. stories = 1, 1, 2, 3]. Finally, a full Combination model also named the Hierarchical Code model, representing the differentiation of semantic, syntactic, and situational processing [pseudowords vs. words vs. sentences vs. stories = 1, 2, 3, 4] was examined. The above weights served as regressors in second-level analyses to separately estimate brain activation for children and adults, while controlling for the effects of age and gender as covariates of no interest in each cohort. In total, we constructed seven regression models in children and adults, respectively, each testing a distinct cognitive hypothesis by identifying brain regions sensitive to either individual cognitive components (semantics, syntax, or situation) or specific combinations of two or three components (Figure 1B). Statistical inference was conducted using a voxel-wise threshold of *p* < 0.001 (uncorrected), with cluster-level family-wise error (FWE) correction applied at *p* < 0.05. Additionally, when projecting the results of brain activation onto the standardized template, we outlined the boundaries of the language network on the brain maps to visualize the activation of each language component in relation to this circuit. This language network was defined following Fedorenko et al. (2010), using a probabilistic overlap map derived from 220 participants who showed greater responses to sentences than to nonword lists.

### Cognitive Model Fitting and Hierarchical Clustering Based on Representational Similarity

In addition to the activation analyses, we conducted RSA to further examine the patterns of neural activity within the language network. Specifically, we employed Fedorenko’s language network, which was derived from a probabilistic overlap map of 220 participants showing greater responses to sentences than to a list of nonwords (Fedorenko et al., 2010). The language network comprises 12 parcels which include three regions in the left frontal cortex (LIFGorb, LIFG, and LMFG) and three regions in the left temporal cortex (LAntTemp, LMidTemp, and LPostTemp), as well as their corresponding regions in the right hemisphere. The RSA was performed on all voxels falling within this a priori language network, regardless of whether their activation exceeded a statistical threshold, to ensure a consistent and comparable set of voxels across all conditions when constructing the inter-condition representational similarity matrix.

We first calculated the representational similarity matrix of each language ROI across conditions (pseudowords, words, sentences, and stories) and then quantified their associations with the seven cognitive models to evaluate the model-ROI fit. To investigate the internal functional structure of the language regions, we further performed clustering analysis among twelve ROIs based on their representational patterns and the fit of seven models.

Specifically, voxel-level representational patterns were first extracted from all 12 language ROIs under the four task conditions (Figure 5A) in each participant. Based on these patterns, a condition-by-condition similarity matrix was computed for each ROI via Pearson correlation. This matrix was further converted into a distance matrix (1 − *r*) and used as the dependent variable for subsequent model-fitting analyses. To quantify the associations between the representational patterns of each language ROI and the seven cognitive models, we calculated the *R*^2^ values in each participant and then averaged them across all participants. The goodness-of-fit was evaluated for each ROI-model pair based on the mean *R*^2^ values.

Then, a hierarchical clustering analysis was further conducted based on the mean model-ROI *R*^2^ values across all participants to reveal the internal organizational principles among language ROIs. A binary tree was generated using average-linkage clustering, illustrating the hierarchical grouping of regions. We then determined the optimal partition by locating the elbow point in the dendrogram, characterized by abrupt changes in branch length and similarity patterns. This resulted in three functional modules in our data: the right hemisphere, left anterior frontotemporal, and left posterior temporal lobes. Last, we investigated developmental differences within the three identified clusters. Specifically, we assessed whether the best-fitting cognitive models for each cluster differed between children and adults.

### Developmental Differences in Model Contributions to Cluster-Level Representational Similarity Patterns

Due to the statistical collinearity among the seven cognitive models (e.g., the combination of the semantic model and the syntactic model forms the semantic + syntactic model, see Figure 1B) and to further identify the models that most significantly predict cluster-level representational patterns, we employed Shapley Additive Explanations (SHAP) analysis to quantify the contributions of all seven models. SHAP values were derived from an Elastic Net regression model, which provides a robust measure of feature importance and is particularly well suited for data exhibiting collinearity (Zou & Hastie, 2005). For each brain cluster and age group (adults and children), we first trained an Elastic Net regression model on the respective data. The SHAP values were then calculated for each model within the trained model. To assess whether the importance of each model was significantly greater than what would be expected by chance, we employed a permutation testing procedure. For each group and cluster, we performed 1,000 permutations. In each permutation, the neural data were randomly shuffled, a new Elastic Net regression model was then trained on this permuted data, and its corresponding SHAP values were computed. The average SHAP values from these 1,000 permutations formed a null distribution. The *p*-value for the original model’s average SHAP value was determined by its position within this null distribution. To account for multiple comparisons across the seven models within each group and cluster, we applied the False Discovery Rate correction to these *p*-values. For validation, we also computed SHAP values using Ridge and Random Forest regression models, all of which yielded consistent results (see Supplementary Figures S3–S5). Considering that SHAP values can effectively quantify the relative contribution of each cognitive model to cluster-level predictions, developmental differences were examined only in models with significant predictive power. Additionally, cluster-level developmental differences may be influenced by neighboring clusters (Chiou et al., 2025). To investigate the unique developmental contributions of each cluster, we further conducted cluster-specific multiple regression analyses. Specifically, for a given cluster, regression models were constructed with the cognitive models predicting the condition-by-condition distance matrix, while controlling for condition-by-condition distance matrix in the other two clusters. This approach isolated the unique explanatory power of each model for the target cluster, removing shared variance attributable to the remaining clusters. For each participant, the *β* coefficients for each cognitive model were extracted as indices of the model’s explanatory power within that cluster. Developmental differences were then assessed by performing independent samples *t*-tests on these cluster-specific *β* coefficients, revealing cognitive models that significantly differ between children and adults in predicting neural patterns.

## RESULTS

### Behavioral Results

Descriptive statistics for both groups are presented in Table 1. The children’s sample consisted of 34 participants (22 males; *M* age = 11.87 years, *SD* = 1.11, range = 9.86–13.88 years), and the adult sample consisted of 30 participants (12 males; *M* age = 23.52 years, *SD* = 2.44, range = 19.00–31.00 years). All participants completed a standardized battery of language and reading assessments. Adults outperformed children on Character Reading Fluency (*M* = 132.72, *SD* = 19.92 vs. *M* = 121.13, *SD* = 18.29) and Verbal Fluency (*M* = 18.21, *SD* = 3.33 vs. *M* = 13.55, *SD* = 4.00). Children showed longer rapid automatized naming times (*M* = 12.29 s, *SD* = 2.87) relative to adults (*M* = 8.79 s, *SD* = 2.21). Importantly, children’s scores on all measures were broadly consistent with performance levels typically reported for this age group in the literature, with individual differences remaining moderate and typical for grades 4–6, suggesting that the sample was reasonably representative of typically developing readers rather than reflecting an unusually wide distribution of reading skill (Supplementary Figure S6).

**Table 1.** Descriptive statistics of behavioral measures for children and adults.

Variables	Children	Adult
	*M* ± *SD* (Range)	*M* ± *SD* (Range)
*N*	34 (Male = 22)	30 (Male = 12)
Age (years)	11.87 ± 1.11 (9.86–13.88)	23.52 ± 2.44 (19.00–31.00)
Rapid Automatized Naming	12.29 ± 2.87 (9.02–19.61)	8.79 ± 2.21 (5.50–14.10)
Character Reading Fluency	121.13 ± 18.29 (88.00–158.00)	132.72 ± 19.92 (95.50–170.00)
Verbal Fluency	13.55 ± 4.00 (8.00–25.49)	18.21 ± 3.33 (11.75–25.25)

*Note*. Rapid automatized naming scores reflect the average time (in seconds) to complete one reading of the number matrix, with longer times indicating slower and less efficient lexical decoding. Behavioral data from one child were missing; therefore, behavioral analyses were conducted on 33 children.

### Univariate Activation Analysis

General activation patterns across language subcomponents were first examined, with detailed results reported in Supplementary Figures S1–S2. In the adult group, activations across all conditions relative to the pseudoword baseline were primarily located within core language network regions, including the left MTG, left inferior frontal gyrus, and bilateral temporal poles. In the children group, activations were similarly concentrated in the bilateral MTG and the cerebellum. Adults showed component-specific activations in the left MTG (sentence > word, capturing syntactic processing) and the right insula and the left postcentral gyrus (story > sentence, capturing situation model processing), whereas children exhibited bilateral MTG (sentence > word) and the left temporal lobe (story > sentence).

### Model-Based Multivariate Regression: Results in Children and Adults

To delineate the neural substrates that subserve the complex cognitive process of language, we constructed seven models capturing cognitive processes from basic linguistic components (semantics, syntax, situation) to their complex combinations (Figure 1B). The Single Code includes three models, each featuring a single component: semantic model, syntactic model, and situation model; The Combination Code also comprises four models, each integrating two components: semantic + syntactic model, semantic + situational model, syntactic and situational model and all three components: semantic + syntactic + situational model (also called hierarchical-code model). We conducted separate model-based multivariate regression analyses for each model in children and adults.

**Figure 2.**
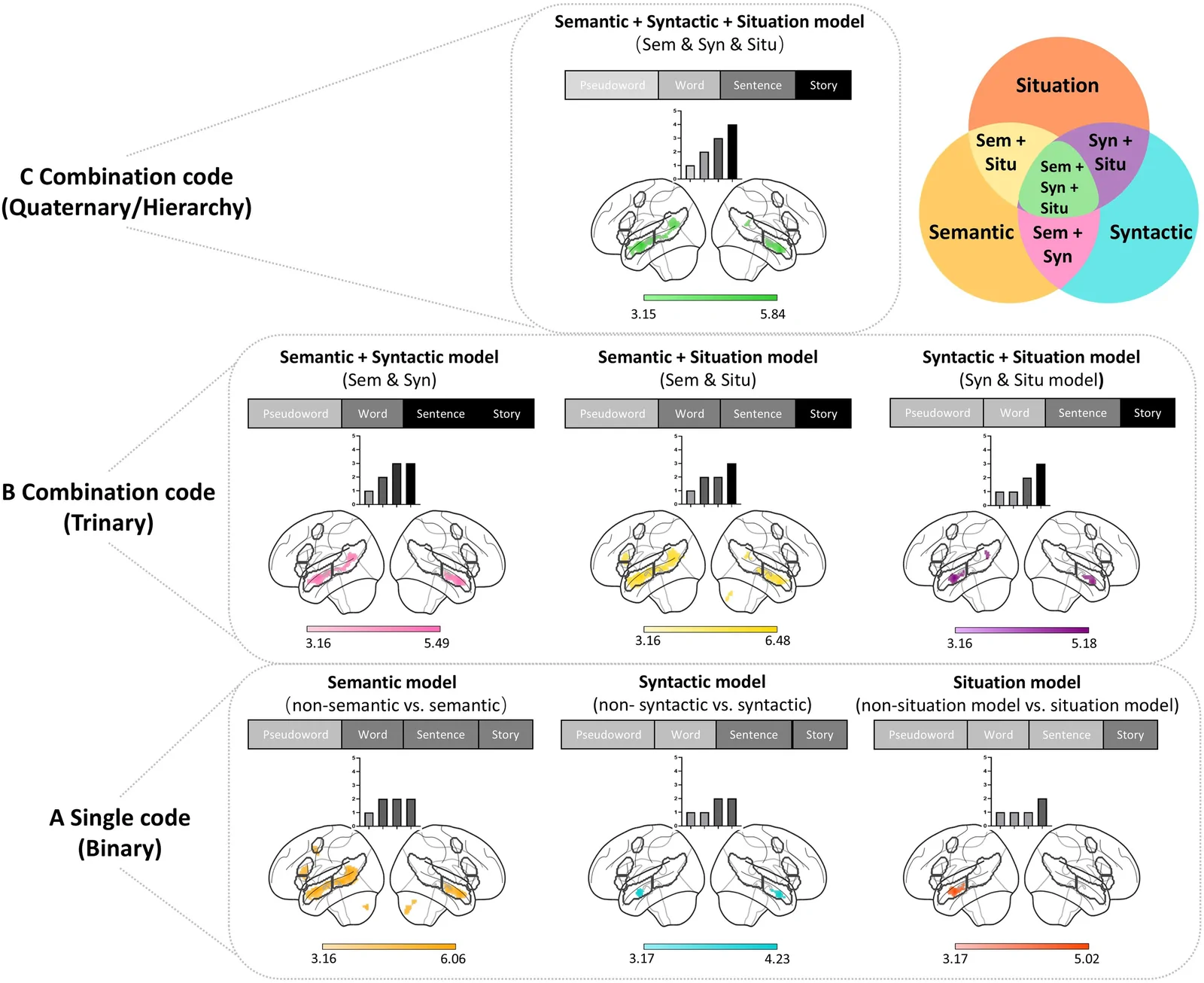
Results of multiple regression analysis in the adult group. (A) Bottom row: Single Code (Binary) models differentiate among the three cognitive components by assigning different weights to linguistic units: semantic (pseudoword condition weight = 1, weights of other linguistic units = 2), syntactic (non-syntactic vs. syntactic: pseudowords and words = 1, sentences and stories = 2), and situational (non-situational vs. situational: pseudowords, words, and sentences = 1, story = 2). (B) Middle row: Combination Code (Trinary) models represent pairwise combinations of two Single Code models: semantic + syntactic, semantic + situational, and syntactic + situational. (C) Top row: The Combination Code (Quaternary / Hierarchical) model captures the hierarchical differentiation of semantic, syntactic, and situational processing. Numerical values represent standardized *β* coefficients from whole-brain multiple regression analysis. Brain activation maps were thresholded at voxel-level *p* < 0.001 (uncorrected) and cluster-level *p* < 0.05 (family-wise error corrected). The outline of the language network shown on the brain maps was defined by Fedorenko et al. (2010). Single Code Models: semantic: Orange, syntactic: Teal, and situation: Red. Combination Code Models: semantic + syntactic: Pink, semantic + situation: Yellow, and syntactic + situation: Purple. semantic + syntactic + situation: Green. Abbreviations: Sem, Semantic; Syn, Syntactic; Situ, Situation.

In adults, activation across all models showed substantial spatial overlap and was predominantly restricted to the left frontal and temporal cortices (see Figure 2). Within the single-code models, the semantic and syntactic models engaged bilateral temporal regions, whereas the situational model was restricted to the left temporal region (Figure 2A). In addition, the semantic model recruited left frontal regions and exhibited more extensive temporal activation. Regarding the combination models, the three pairwise models (semantic + syntactic, semantic + situational, and syntactic + situational) commonly activated bilateral temporal regions, with the semantic + situational model uniquely extending into the left frontal region (Figure 2B). In contrast, the full combination model (semantic + syntactic + situational model) showed activation primarily confined to bilateral temporal regions (Figure 2C).

**Figure 3.**
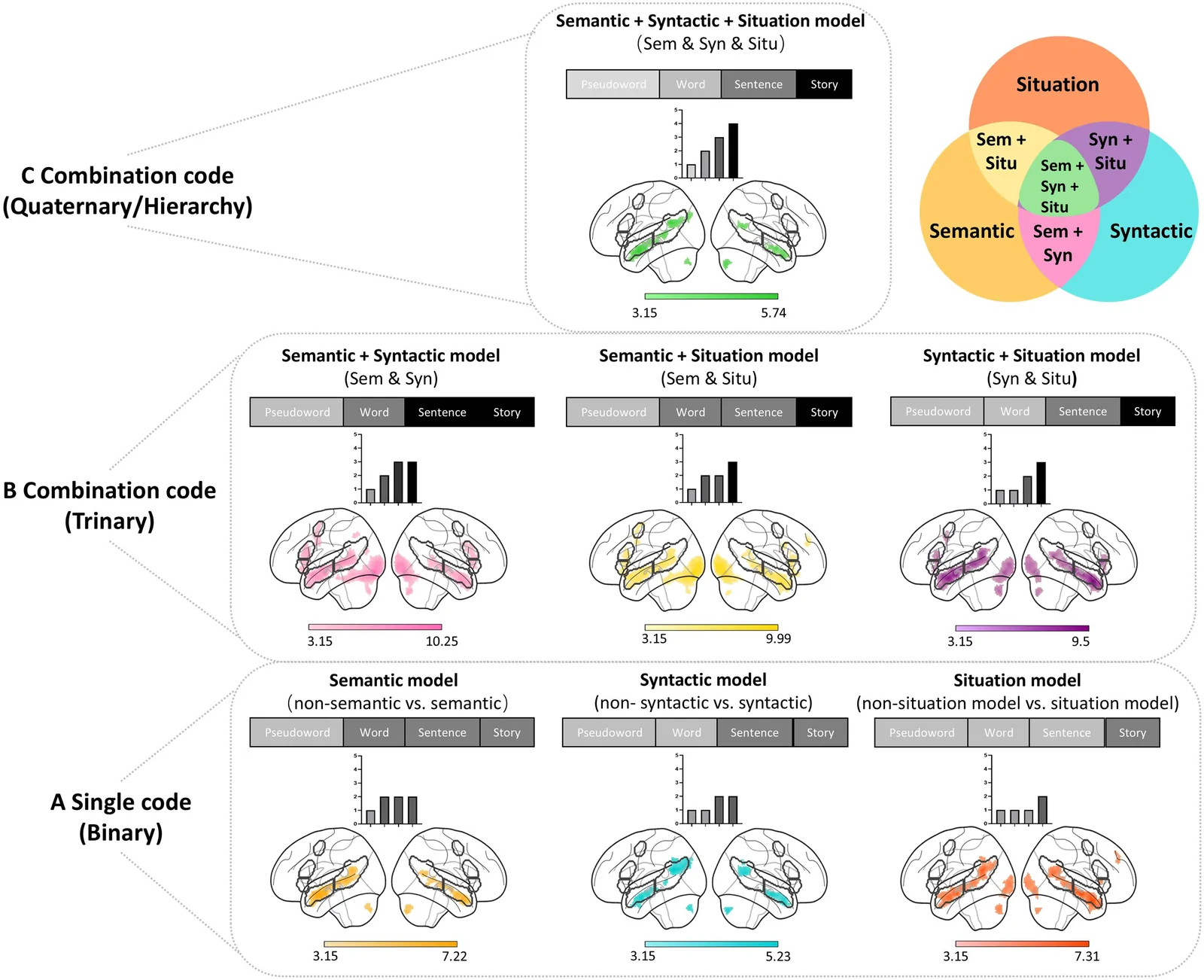
Results of multiple regression analysis in the child group. (A) Bottom row: Single Code (Binary) models differentiate among the three cognitive components by assigning different weights to linguistic units: semantic (pseudoword condition weight = 1, weights of other linguistic units = 2), syntactic (non-syntactic vs. syntactic: pseudowords and words = 1, sentences and stories = 2), and situational (non-situational vs. situational: pseudowords, words, and sentences = 1, story = 2). (B) Middle row: Combination Code (Trinary) models represent pairwise combinations of two Single Code models: semantic + syntactic, semantic + situational, and syntactic + situational. (C) Top row: The Combination Code (Quaternary / Hierarchical) model captures the hierarchical differentiation of semantic, syntactic, and situational processing. Numerical values represent standardized *β* coefficients from whole-brain multiple regression analysis. Brain activation maps were thresholded at voxel-level *p* < 0.001 (uncorrected) and cluster-level *p* < 0.05 (family-wise error corrected). The outline of the language network shown on the brain maps was defined by Fedorenko et al. (2010). Single Code Models: semantic: Orange, syntactic: Teal, and situation: Red. Combination Code Models: semantic + syntactic: Pink, semantic + situation: Yellow, and syntactic + situation: Purple. semantic + syntactic + situation: Green. Abbreviations: Sem, Semantic; Syn, Syntactic; Situ, Situation.

In the child group, the three single-code models (semantic, syntactic, and situation models) all engaged the bilateral temporal regions and cerebellum. Additionally, both the syntactic and situational models activated the left precuneus, whereas the situational model further recruited the bilateral middle occipital gyrus and right frontal regions (Figure 3A). The three pairwise combination models (semantic + syntactic model, semantic + situational model, and syntactic + situational model) engaged a broader network, commonly involving the bilateral temporal and occipital regions, frontal regions, and cerebellum (Figure 3B). In contrast, the full combination model (semantic + syntactic + situational model) showed a relatively more restricted activation pattern, primarily confined to the bilateral temporal regions and cerebellum (Figure 3C).

### Model-Based Multivariate Regression: Children Versus Adults

**Figure 4.**
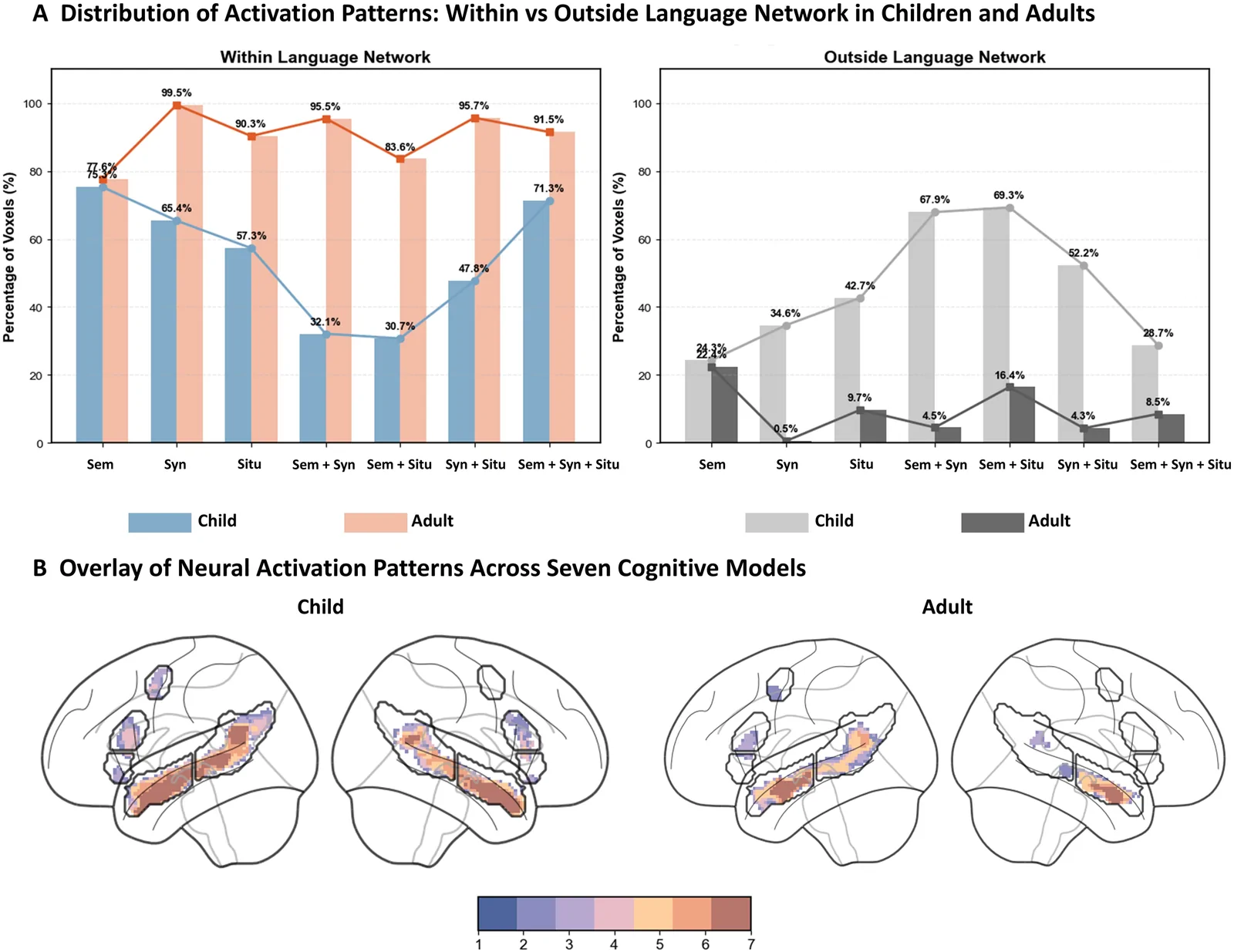
Spatial distribution of neural activation patterns across cognitive models in children and adults. (A) Bar and line graphs depict the percentage of voxels (%) associated with activation patterns related to the seven cognitive models, both within (blue/orange bars and lines) and outside (gray/black bars and lines) the language network for children and adults. (B) Overlay of neural activation patterns for children and adults across the seven cognitive models, illustrating the spatial distribution of brain activity. Within the language network, adults exhibit a more restricted activation pattern compared to children, which may reflect a more fine-tuned representational organization. Abbreviations: Sem, Semantic; Syn, Syntactic; Situ, Situation.

To examine developmental differences in language network recruitment in processing different linguistic components, we first quantified the proportion of activated voxels within the language network for each cognitive model. In children, Single Code models showed high within-network proportions (Semantic: 75.7%; Syntactic: 65.4%; Situation: 57.3%), whereas three pairwise Combination Code models exhibited markedly lower proportions (Semantic + Syntactic: 32.1%; Semantic + Situation: 30.7%; Syntactic + Situation: 47.8%). Notably, the full combination model (also called Hierarchical-Code model) maintained a high within-network proportion (71.3%). In adults, high within-network engagement was observed across all models, including Single Code (Semantic: 77.6%; Syntactic: 99.5%; Situation: 90.3%), pairwise Combination Code (Semantic + Syntactic: 95.5%; Semantic + Situation: 83.6%; Syntactic + Situation: 95.7%), and the full Combination/Hierarchical-Code model (91.5%) (Figure 4A). The lower within-network proportions in children’s pairwise Combination Code models, in contrast to adults, suggest that developmental maturation is associated with a more focal recruitment of the core language network, with younger children engaging extra-network regions during complex linguistic processing. Furthermore, the consistently high engagement of core language regions in the full Combination/Hierarchical-Code model indicates that the concurrent integration of multiple linguistic features relies predominantly on the canonical language network in both children and adults.

Figure 4B illustrates the overlay of neural activation patterns for all seven cognitive models in children and adults. Compared to children, adults exhibit a more restricted spatial extent of activation within the language network. This focal activation pattern suggests a more refined neural representation for language processing in the mature brain. Specifically, adult activation is concentrated in fewer core language regions, whereas children engage a broader set of distributed brain areas during linguistic tasks.

### Cluster Organization of the Language Network Based on Cognitive Model Fit

**Figure 5.**
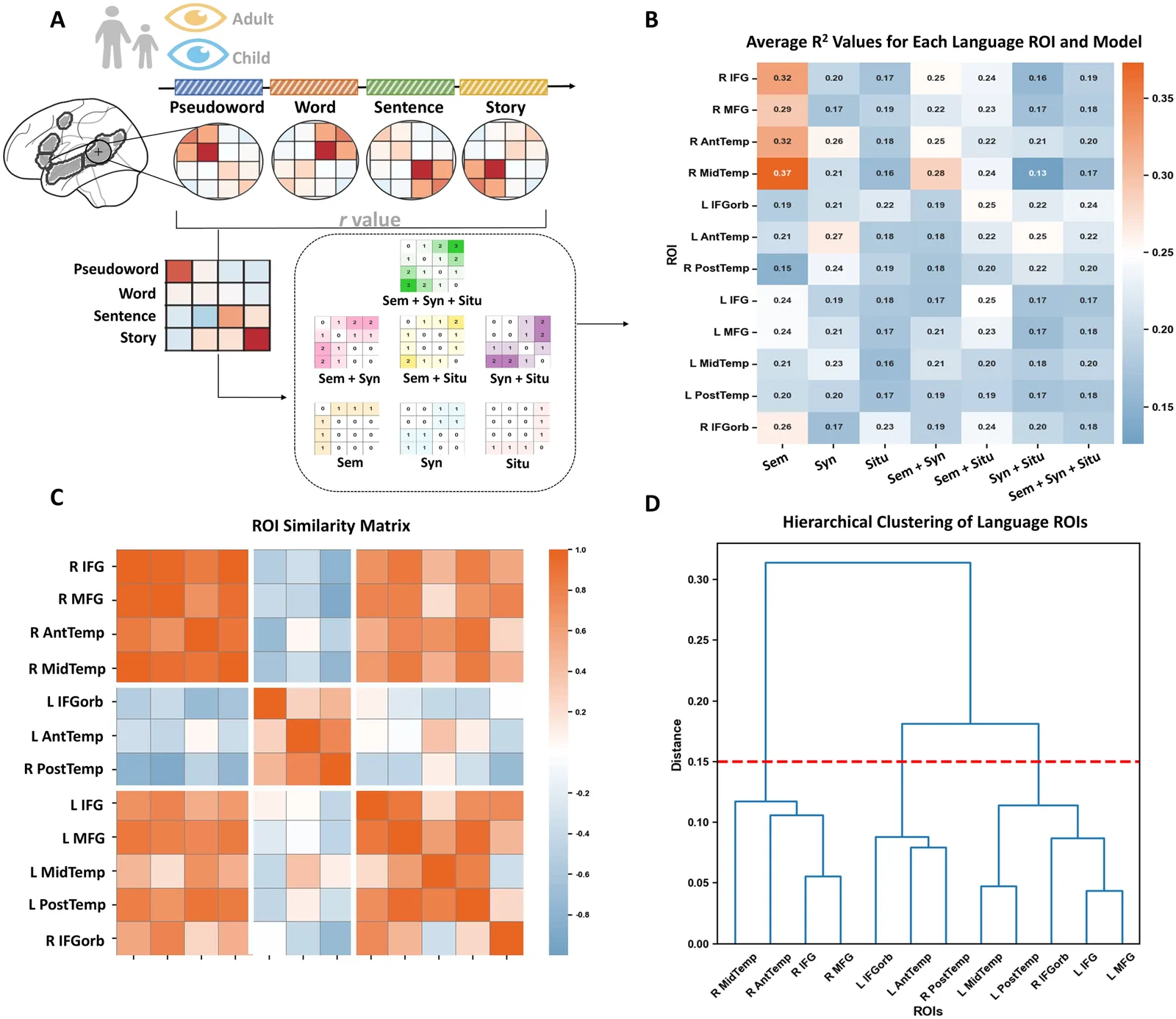
Panel A illustrates the representational patterns of the language network extracted from each participant during reading four conditions (pseudowords, words, sentences, and stories). The representational patterns across conditions were correlated to generate a condition-by-condition distance matrix (1–r). The matrix was then fitted to seven cognitive models to assess how well each model explained the representational structure of linguistic components in the language network. Panel B displays a heatmap of the average *R*^2^ values across language regions and models in each participant. The *y*-axis represents the 12 language ROIs, while the *x*-axis represents the seven models. *R*^2^ values are indicated by both color intensity and numerical labels, where warmer colors correspond to higher *R*^2^ values, indicating stronger model fit. Panel C presents the pattern similarity matrix among the 12 language ROIs within the language network. We found that some of the language ROIs exhibited similar representational patterns, providing the foundation for subsequent hierarchical clustering analysis. Panel D shows a hierarchical clustering dendrogram of the language ROIs, derived from the combined data of children and adults to ensure a common clustering solution for subsequent group comparisons. This clustering was based on the pattern of average *R*^2^ values across the seven models for each brain region. The red dashed line marks the threshold used to partition the ROIs into three main clusters. Abbreviations: Sem: Semantic; Syn: Syntactic; Situ: Situation.

First, the neural patterns of the 12 ROIs in the language network were extracted under the four conditions, and the similarity between conditions was calculated, resulting in a condition-by-condition matrix. We fitted the seven cognitive models to the matrix to determine the optimal one for each language ROI, as assessed based on the *R*^2^ values (Figure 5A). The results indicated a dissociation in model fit among twelve ROIs: the semantic model best explained by the patterns in five right-hemisphere ROIs (right IFG, bilateral MFG, right ATL, right MTG, and right IFG orb) and the left MFG. In contrast, the syntactic model was the best fit for several temporal regions, including the left ATL, bilateral posterior temporal gyrus, and the left MTG (Figure 5B). This finding points to a fundamental organizational principle within the language network. Accordingly, a data-driven hierarchical clustering analysis was conducted to further reveal the internal functional architecture of the language network.

**Figure 6.**
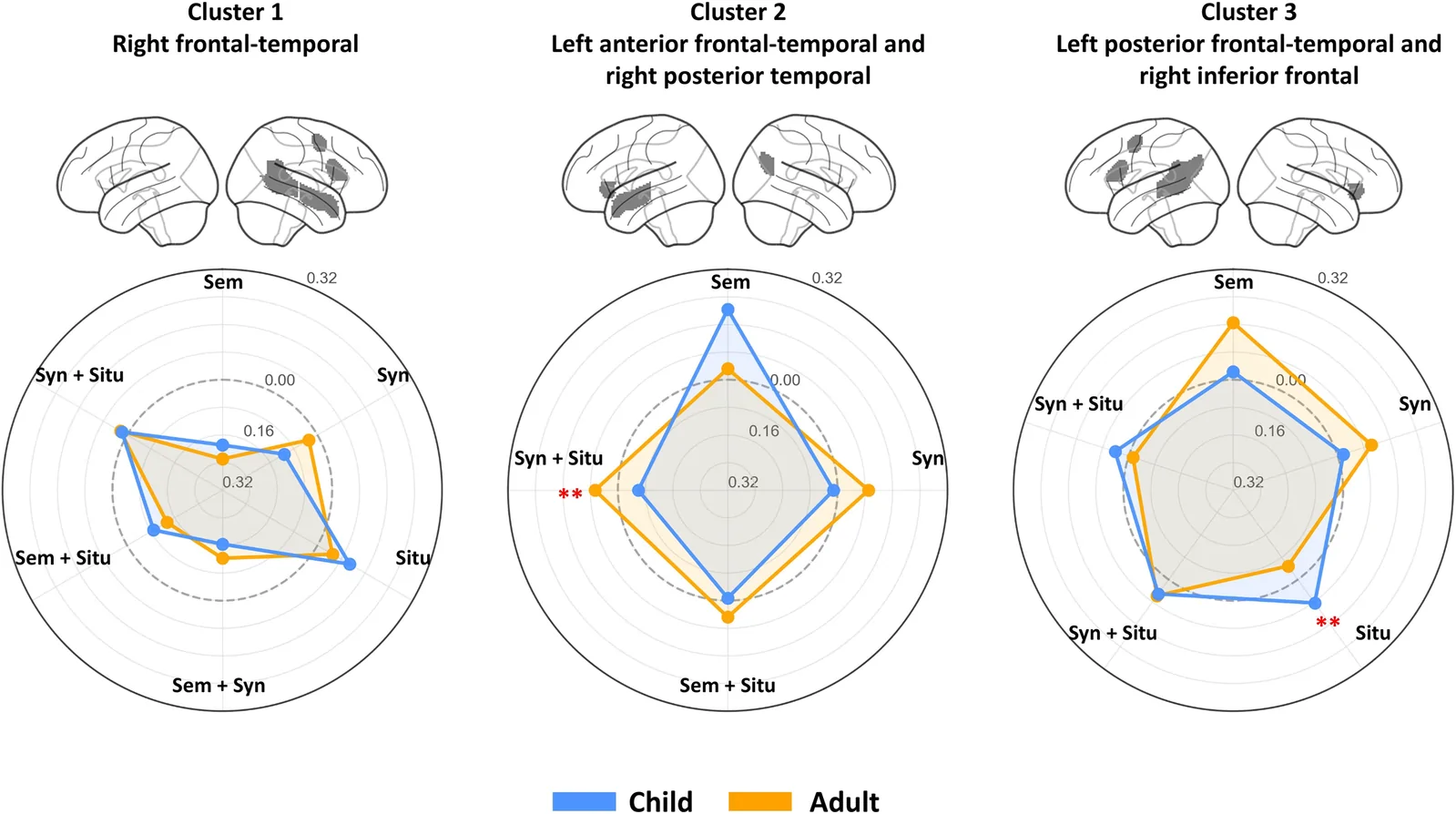
Result of Developmental Differences in Model Contributions. First, brain maps depict the three clusters. Then, radar plots illustrate standardized regression coefficients (*β* values) obtained from multiple regression analyses of models identified as significant based on SHAP values, controlling for the effects of the other two clusters. Orange lines represent adults and blue lines represent children. Red asterisks indicate significant group differences (*p < 0.05, **p < 0.01, ***p < 0.001). Abbreviations: Sem: Semantic; Syn: Syntactic; Situ: Situation.

A tripartite cluster structure emerged from the hierarchical clustering, marked by higher pattern similarity within clusters than between them, thereby delineating the internal functional architecture (Figure 5C–5D). The first cluster, primarily comprising regions on the right hemisphere, included the right middle temporal, right anterior temporal, right inferior frontal, and right middle frontal regions. The second cluster, which corresponds to the frontal and anterior temporal regions, included the left orbital inferior frontal, left anterior temporal, and right posterior temporal regions. The third cluster, involving the frontal and posterior temporal regions, encompassed the left middle temporal, left posterior temporal, right orbital inferior frontal, left inferior frontal, and left middle frontal regions (see Figure 6).

### Results of Cluster-Specific Developmental Differences in Model Contributions

We first assessed the contributions of the cognitive models to the cluster-level representational similarity patterns using SHAP analysis. Based on this analysis, we selected models that exhibited significant SHAP importance in either children or adults for subsequent developmental comparisons. For Cluster 1, SHAP analysis revealed significant contributions from semantic model (Adult: *p* < 0.01, Cohen’s *d* = 20.33), syntactic model (Adult: *p* = 0.02, Cohen’s *d* = 5.94; Child: *p* < 0.01, Cohen’s *d* = 9.69), situation model (Child: *p* < 0.01, Cohen’s *d* = 13.98), semantic + syntactic model (Child: *p* < 0.01, Cohen’s *d* = 21.05), the semantic + situation model (Adult: *p* = 0.01, Cohen’s *d* = 13.03; Child: *p* = 0.0047, Cohen’s *d* = 7.07), and syntactic + situation model (Adult: *p* < 0.01, Cohen’s *d* = 12.19; Child: *p* = 0.0047, Cohen’s *d* = 7.07) in at least one age group. Therefore, these six models were included to examine developmental differences in Cluster 1.

For Cluster 2, SHAP analysis revealed significant contributions from semantic model (Adult: *p* < 0.01, Cohen’s *d* = 6.90), syntactic model (Adult: *p* < 0.01, Cohen’s *d* = 13.69; Child: *p* < 0.01, Cohen’s *d* = 13.29), the semantic + situation model (Adult: *p* < 0.01, Cohen’s *d* = 9.55; Child: *p* < 0.01, Cohen’s *d* = 12.98), and syntactic + situation model (Adult: *p* < 0.01, Cohen’s *d* = 11.83) in at least one age group. Therefore, these four models were included to examine developmental differences in Cluster 2.

For Cluster 3, SHAP analysis revealed significant contributions from semantic model (Adult: *p* = 0.02, Cohen’s *d* = 4.50), syntactic model (Adult: *p* < 0.01, Cohen’s *d* = 18.04; Child: *p* < 0.01, Cohen’s *d* = 12.60), situation model (Child: *p* = 0.03, Cohen’s *d* = 6.78), the semantic + situation model (Adult: *p* < 0.01, Cohen’s *d* = 13.03; Child: *p* = 0.01, Cohen’s *d* = 16.81), and the syntactic + situation model (Adult: *p* < 0.01, Cohen’s *d* = 10.43) in at least one age group. Therefore, five models were included to examine developmental differences in Cluster 3.

To investigate developmental differences in model contributions to Cluster-level representational patterns while avoiding collinearity among neighboring regions (Chiou et al., 2025), we constructed additional regression models controlling for the effects of the other two clusters when evaluating each target cluster. The results demonstrated that, in Cluster 2, adults showed significantly stronger model fit than children for the combination code model of syntactic + situation even after controlling for the other clusters. Conversely, in Cluster 3, children exhibited significantly stronger model fit than adults for the single code model of situation once the contributions of other clusters were partialled out (Figure 6).

## DISCUSSION

This study investigates how the brain processes multiple linguistic subcomponents (specifically via single-component or combination-based coding mechanisms), as well as the functional organization and developmental refinement of the language network. We employed a hierarchical reading paradigm spanning pseudowords, words, sentences, and stories, and defined seven cognitive models to capture these coding patterns: three single-code models (representing individual components of semantics, syntax, and the situation model) and four combinational models (integrating components pairwise or all three together). First, model-based univariate activation analyses revealed broadly overlapping engagement of left temporal and frontal regions across all models in both adults and children, suggesting multifunctional regions that support combination-based coding. Activation in adults was generally more focal and primarily restricted to core language regions across all seven models, whereas children recruited more widespread brain regions both within and beyond the language network (Figures 2–4). These findings suggest a shared representational architecture for language processing that becomes increasingly spatially focal from childhood to adulthood, with adult patterns showing correspondence with a wider range of component and combination models within a more restricted set of regions. Next, we characterized the functional profiles within the language network by performing a data-driven clustering analysis based on the representational similarity patterns of language regions and their model-fitting profiles across the seven cognitive models. The language network was organized into three distinct clusters (Figure 5): Cluster 1, primarily in the right hemisphere, included the right middle temporal, anterior temporal, and inferior/middle frontal regions. Cluster 2 mainly comprised left anterior frontal and temporal regions, along with the right posterior temporal region. Cluster 3 covered the posterior frontal and temporal regions, including the left middle/posterior temporal and left inferior/middle frontal regions. Multivariate regression analyses further revealed significant developmental differences in how these clusters correspond to the cognitive models. In adults, Cluster 2 (anterior fronto-temporal regions) exhibited a more refined processing pattern, showing a significantly stronger correspondence with the Combination Code model of syntactic and situation (syntactic + situation model) compared to children. In contrast, Cluster 3 in children (posterior fronto-temporal regions) was more strongly explained by the Single Code model of situation (situation model) relative to adults (Figure 6). Overall, these results demonstrated that the language network integrated multiple linguistic components through combination-based coding and was internally organized into spatially segregated, functionally homogeneous clusters whose functional roles underwent developmental change. Specifically, adults increasingly integrate complex informational representations within the anterior fronto-temporal network, whereas children rely more heavily on posterior regions to process individual, single-code dimensions of language. This study uncovers the internal functional architecture of the language network and provides novel mechanistic insights into how this fine-grained organization matures for efficient hierarchical language processing.

### Developmental Reorganization: From Whole-Brain Distributed Activation to Focal Recruitment Within the Language Network

In adults, language processing is characterized by a high degree of functional specialization and neural efficiency, primarily localized within a left-lateralized fronto-temporal network (Fedorenko et al., 2011; Mesulam, 2016; Vigneau et al., 2006). Model-based analyses reveal substantial overlap in neural representations across linguistic subcomponents within core language regions, with multiple linguistic dimensions integrated within shared cortical circuits to support efficient compositional operations (Blank & Fedorenko, 2020; Fedorenko et al., 2020; Fusi et al., 2016; Hagoort & Indefrey, 2014; Rigotti et al., 2013). Thus, the adult language system achieves processing efficiency through converging multiple linguistic computations within a compact, highly selective cortical architecture.

In contrast, children exhibit activation patterns extending beyond the core language network, encompassing bilateral occipital cortices and cerebellar regions, reflecting additional recruitment of domain-general regions during early skill acquisition (Benischek et al., 2020; Esver et al., 2025; Gaillard et al., 2003; Johnson, 2011; Knops et al., 2009; Mechtenberg et al., 2024; Qi et al., 2021; Wagner et al., 2025; Weiss-Croft & Baldeweg, 2015). Within the fronto-temporal network specifically, frontal engagement primarily reflects integration across linguistic subcomponents, while posterior temporal regions show widespread bilateral activation supporting situational model construction and discourse-level integration (Schmithorst et al., 2006; Szaflarski et al., 2012). Notably, because the present study employed a visual reading paradigm, prior research has demonstrated that the broader recruitment of occipital and fusiform regions in children may be more susceptible to differences in reading fluency and orthographic decoding (Benjamin & Gaab, 2012; Chen et al., 2019; Dehaene-Lambertz et al., 2018; Langer et al., 2015; Martin et al., 2015; Monzalvo & Dehaene-Lambertz, 2013; Siok et al., 2020; Wu et al., 2012). In contrast, our primary findings within the fronto-temporal language network are likely to be less affected by such basic decoding differences, as studies have shown that this network largely supports higher level linguistic processing that operates relatively independently of visual decoding efficiency and general cognitive load (Schlaggar & McCandliss, 2007; Scott et al., 2017).

Collectively, these patterns reveal a developmental shift from a distributed, resource-intensive neural strategy in childhood to a streamlined, specialized system in adulthood, consistent with an automatization account (Friederici, 2011): increasing linguistic expertise drives the brain away from reliance on auxiliary recruitment toward more efficient combinatorial coding within specialized fronto-temporal circuits (Mill & Cole, 2025; Weiss-Croft & Baldeweg, 2015; Wu et al., 2024).

### Functional Organization of the Language Network: Clustering Evidence for Lateralization and Anterior–Posterior Specialization

Unlike prior studies that examined the frontal and temporal lobes in isolation (Chai et al., 2016; Geng et al., 2023; Mesulam, 2016; Morishima et al., 2025; Ojemann, 1991; Spitsyna et al., 2006; Stone et al., 2025; Wang et al., 2021; Wilson, 2017), our clustering analysis revealed that cluster 1 predominantly encompasses ROIs within the right-hemisphere language network, whereas the anterior portion of the left fronto-temporal network forms a distinct cluster (Cluster 2), and the posterior portion constitutes another separate cluster (Cluster 3). The functional distinction between Cluster 2 and Cluster 3 is reflected in a hierarchical processing along the anterior-posterior axis, supporting the processing of language subcomponents. In the cluster analyses of the language network, although the developmental differences did not reach statistical significance, the developmental pattern of Cluster 1 indicated that children were better characterized by models combining semantic and syntactic processes (i.e., semantic + syntactic model), whereas adults were more aligned with models emphasizing semantic processing (i.e., semantic model). These findings suggest that, in children, representational patterns in right-hemisphere language regions show stronger correspondence with models capturing both semantic and syntactic information. This pattern provides initial evidence that representational coding in the right hemisphere becomes more selective with development.

The developmental trends in Cluster 1 can be interpreted in light of the protracted lateralization and specialization of the language network (Asaridou et al., 2020; Berl et al., 2014; Prat et al., 2023; Wagley & Booth, 2021). Previous developmental studies suggest that language processing is relatively more bilateral in children than in adults, with right-hemisphere involvement decreasing with age despite an early-emerging left-hemisphere bias (Berl et al., 2014; Olulade et al., 2020; Szaflarski et al., 2006; Wagley & Booth, 2021). In parallel, semantic and syntactic computations become progressively differentiated, with syntactic processing showing prolonged specialization in left frontotemporal regions and gradual segregation from semantic processing during late childhood and early adolescence (Fedorenko et al., 2020; Law & Pylkkänen, 2021; Matchin & Hickok, 2020; Nuñez et al., 2011; Rodd et al., 2015; Siegelman et al., 2019; Skeide et al., 2014; Skeide & Friederici, 2016). Within this framework, the trend toward stronger correspondence with the semantic + syntactic model in children may tentatively reflect residual right-hemisphere involvement in both semantic and syntactic information (Vigneau et al., 2011), whereas adults’ tendency toward the semantic model aligns with a reduced right-hemisphere contribution to syntax (Kauf et al., 2024). Although these differences did not reach statistical significance, Cluster 1 offers a suggestive, RSA-based indication of a developmental shift from broad bilateral language engagement toward a more specialized and left-lateralized language architecture.

The RSA results for Clusters 2 and 3 reveal a developmental shift from syntactically anchored coding toward semantically centered, component-specific representations (Fedorenko et al., 2020; Matchin & Hickok, 2020; Rodd et al., 2015; Siegelman et al., 2019). In children, both clusters were broadly characterized by combinational models of semantic and situational information alongside the syntactic Single Code model, without clear semantic differentiation, whereas adults showed more specialized patterns corresponding to distinct Single Code models for semantic and syntactic processing, alongside combination models integrating situational information (Supplementary Figure S3). This developmental divergence suggests that adult language processing may undergo a semantic-centered reorganization (Martin et al., 2023), supporting the concurrent integration of lexical-contextual information. In contrast, children do not exhibit clearly differentiated representations of lexical and contextual information, relying more heavily on syntactic structures to support comprehension (Hahne et al., 2004; Law & Pylkkänen, 2021; Schneider & Maguire, 2019). The distinction between Clusters 2 and 3 is supported by converging evidence for anterior–posterior functional specialization within the fronto-temporal network. Previous studies have shown that the anterior and posterior portions of the frontal and temporal lobes correspond to distinct functional roles. Moreover, these findings align with the notion that neural activity propagates sequentially across brain regions in a pulse-like manner, dynamically reorganizing cortical network interactions and supporting a “topographic” model of information flow, whereby information is transmitted along the anterior-posterior axis in a structured temporal sequence (e.g., Mohan et al., 2024; Xu et al., 2023). Together, this body of evidence provides an anatomical basis for understanding the developmental representational differences observed between Clusters 2 and 3.

It is important to note that many previous studies by Fedorenko and colleagues have excluded the bilateral angular gyrus from the language network (e.g., Fedorenko et al., 2024). In the present study, the bilateral angular gyrus was included because univariate analyses showed that its activation patterns, particularly in children, could be accounted for by the syntactic model, situation model, and Hierarchy Code cognitive models, suggesting its potential relevance to developmental differences in language representational patterns (Acunzo et al., 2022). Therefore, we retained the bilateral angular gyrus to explore developmental effects on representational patterns. Furthermore, in the clustering analyses, the left angular gyrus was included in Cluster 3 and the right angular gyrus in Cluster 2, indicating that their representational profiles were similar to those of other core language network regions rather than forming a separate cluster.

### Anterior–Posterior Dissociation in Situation Model Representation: Developmental Reorganization From Posterior Scaffolding to Anterior Integration

Our results showed that Cluster 2 (anterior fronto-temporal regions) exhibited a more refined processing pattern, demonstrating stronger correspondence with syntactic + situation model in adults compared to children (Caucheteux et al., 2023; Ding et al., 2016; Ferstl et al., 2008; Lerner et al., 2011). In contrast, Cluster 3 in children (posterior fronto-temporal regions) showed stronger correspondence with situation model relative to adults (Figure 6). Studies suggest that the ATL, particularly in the left hemisphere, exhibits stronger activation during syntactic processing (Brennan et al., 2012; Friederici, 2011; Humphries et al., 2005, 2006; Matchin & Hickok, 2020; Persichetti et al., 2021; Vandenberghe et al., 2002), and preferentially supports situation models through its integration with syntactic representations, contributing to the construction of coherent, context-rich representations that extend beyond word-level meaning (Acunzo et al., 2022; Brennan et al., 2012; Fedorenko et al., 2016; Ferstl et al., 2008; Hagoort, 2005; Lerner et al., 2011; Tuckute et al., 2022; Vandenberghe et al., 2002). This body of evidence helps explain why, relative to children, adults show more pronounced syntactic and situation model representations in anterior fronto-temporal regions. Importantly, this anterior–posterior dissociation provides novel evidence that situation-related representations, operationalized here as discourse-level integration demands, show a developmentally reorganized representational profile within the language network, rather than merely emerging as a by-product of semantic or syntactic processing (Ivanova et al., 2021).

**Figure 7.**
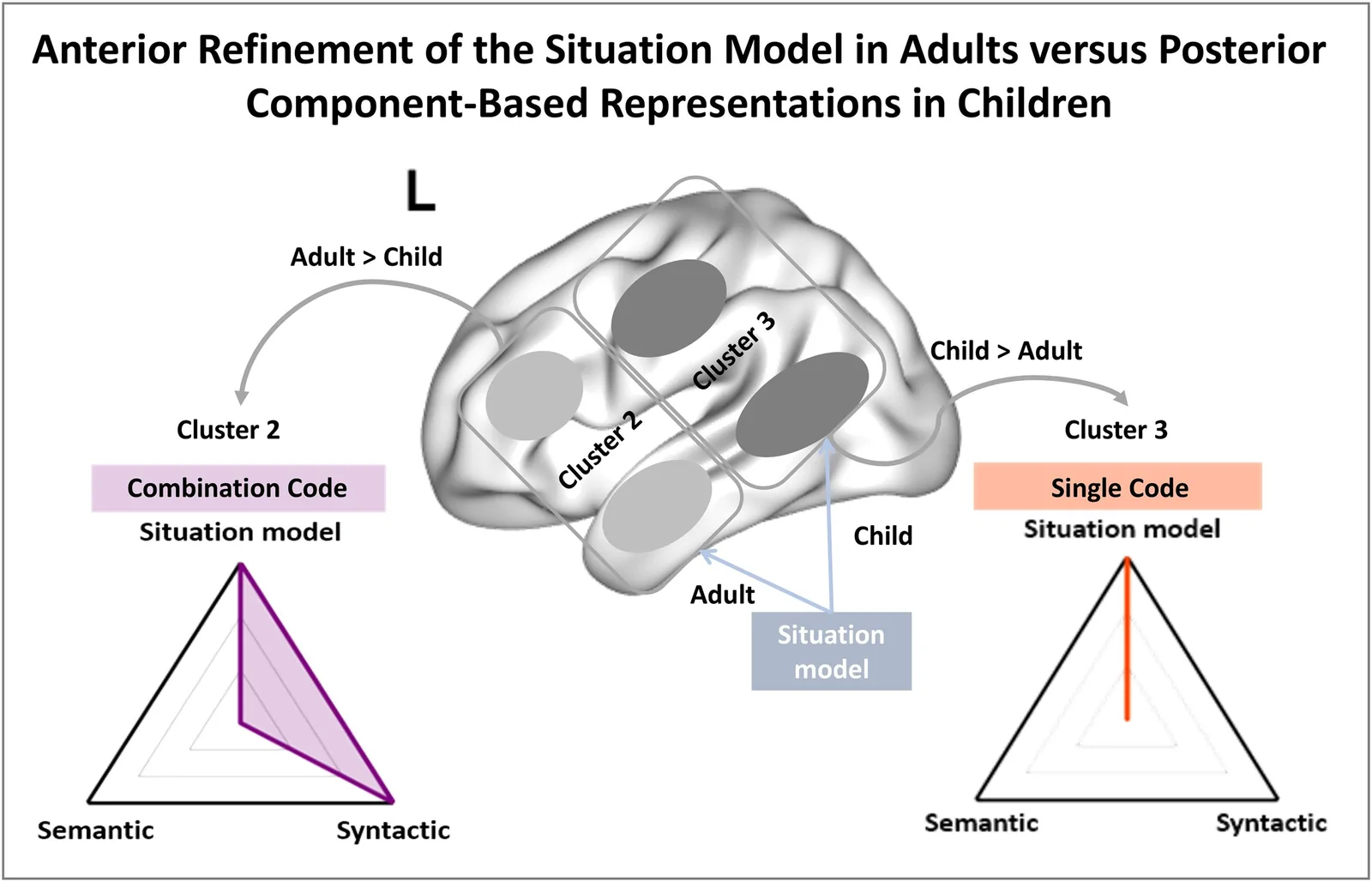
Developmental Reorganization of Situation-Model Representations: Anterior Refinement in Adults and Posterior Component-Based Patterns in Children. Our results showed that Cluster 2 (anterior fronto-temporal regions) exhibited a more refined processing pattern, demonstrating stronger correspondence with Combination Code (i.e., syntactic + situation) in adults compared to children. In contrast, Cluster 3 in children (posterior fronto-temporal regions) showed stronger correspondence with Single Code (i.e., situation) relative to adults.

In children, however, the situation model is primarily differentiated in posterior temporal regions, reflecting a representational organization of single code that is more pronounced than in adults (Figure 7). This difference may reflect the protracted maturation of the anterior temporal lobe, as structural and functional development in this region, including processes such as myelination and synaptic pruning, continues into adolescence (Liang et al., 2024; Petanjek et al., 2011). This developmental delay limits children’s ability to form fine-grained representations and to perform high-level integrative processing. As a result, posterior temporal regions act as a compensatory scaffold in children, supporting situational processing (Acunzo et al., 2022; Skeide et al., 2016). With development, the anterior temporal lobe in adults becomes a central hub for efficient combinatorial processing, capturing finer-grained syntactic and situational information (Persichetti et al., 2021).

### Limitations and Future Directions

Several limitations of this study should be acknowledged. First, although comparable to prior developmental fMRI studies, the sample size was relatively modest and restricted to typically developing Mandarin-speaking children and young adults. This limits the generalizability of our findings across broader populations, such as children with reading difficulties, speakers of other languages, or different cultural contexts (Geng et al., 2023; Zhu et al., 2014). Second, the cross-sectional design precludes firm conclusions about developmental trajectories; longitudinal studies will be necessary to establish causal patterns of neural reorganization. Third, despite using age-appropriate materials and a natural reading paradigm to minimize task demands, the visual reading modality may still introduce confounds related to reading fluency and attentional demands. Because children read less fluently than adults, basic decoding imposes higher cognitive and attentional demands on them, raising the possibility that observed developmental differences partly reflect differences in cognitive resource allocation rather than linguistic processing per se. Future studies should therefore employ an auditory paradigm (e.g., free listening) using the same stimuli as the current study. This would help disentangle reading fluency and attentional demands from general language processing, and clarify whether the developmental differences observed here extend to auditory language comprehension. In addition, future studies should incorporate in-scanner comprehension probes to provide direct, real-time verification of task engagement, thereby addressing the absence of concurrent behavioral engagement measures in the present study. Fourth, although the reading-related skills of the child sample showed a relatively concentrated distribution with limited individual variability, we cannot entirely rule out the possibility that the remaining individual differences in these abilities may partially contribute to the broader and more variable neural activation patterns observed in children. Future studies should incorporate brain-behavior correlation analyses to more directly examine the relationship between reading skill and neural activation patterns. Finally, although this study revealed fine-grained developmental differences in the representations of distinct clusters within the language network, future research should examine whether these clusters extend to whole-brain connectivity and whether such connectivity demonstrates consistent functional specificity (Morishima et al., 2025; Rolls et al., 2022).

## CONCLUSION

This study investigates whether the language network integrates multiple linguistic components via regions specialized for individual components (single-component coding) or via regions representing combinations of components (combination-based coding), and how these representational patterns differ between children and adults. The results indicated that, across seven cognitive models representing combinations of language subcomponents, children recruited more widespread brain networks associated with these models, extending beyond the core language regions, whereas adults exhibited more specialized activation largely confined to the canonical language network. Moreover, data-driven clustering analysis based on the representational association patterns between the language network and seven cognitive models revealed three distinct clusters, reflecting the internal organizational principles of the network. Finally, significant group differences were observed in how the cognitive models contributed to the cluster patterns. Compared to children, adults’ anterior fronto-temporal regions (Cluster 2) were more strongly driven by Combination Code model of syntactic and situation (syntactic + situational model), whereas children engaged posterior temporal regions (Cluster 3) more extensively for Single-code model of situational processing (situational model). In addition, within Cluster 1, a non-significant trend was observed whereby children showed relatively stronger correspondence with the Combination Code model of semantic and syntactic processing (semantic + syntactic model), whereas adults showed relatively stronger correspondence with the Single Code model of semantic processing. Overall, these results provide evidence that representational patterns in the language network are consistent with combination-based coding, highlighting how it evolves from a broadly distributed system in childhood to a more specialized and efficiently integrated network in adulthood. This study provides novel mechanistic insights into how the language network represents semantic, syntactic, and situational information and establishes a foundation for future investigations of the neural mechanisms supporting efficient hierarchical language processing.

## FUNDING INFORMATION

Xiangzhi Meng, National Natural Science Foundation of China (https://dx.doi.org/10.13039/501100001809), Award ID: 32371141. Xiaoxia Feng, National Natural Science Foundation of China (https://dx.doi.org/10.13039/501100001809), Award ID: 32400893. Xiujie Yang, National Natural Science Foundation of China (https://dx.doi.org/10.13039/501100001809), Award ID: 32571246. Guosheng Ding, National Natural Science Foundation of China (https://dx.doi.org/10.13039/501100001809), Award ID: 32471100. Xiujie Yang, Fundamental Research Funds for the Central Universities (https://dx.doi.org/10.13039/501100012226), Award ID: 1252000746. Xiaoxia Feng, Fundamental Research Funds for the Central Universities (https://dx.doi.org/10.13039/501100012226), Award ID: 2263100008.

## AUTHOR CONTRIBUTIONS

**Jie Chen**: Conceptualization: Lead; Data curation: Equal; Formal analysis: Lead; Investigation: Equal; Visualization: Lead; Writing – original draft: Lead; Writing – review & editing: Equal. **Xiaoxia Feng**: Conceptualization: Equal; Visualization: Supporting; Writing – review & editing: Lead. **Jia Zhang**: Data curation: Lead; Writing – review & editing: Supporting. **Chan Tang**: Investigation: Supporting; Writing – review & editing: Supporting. **Linyan Liu**: Conceptualization: Supporting; Investigation: Supporting; Visualization: Supporting. **Siyi Fan**: Writing – review & editing: Supporting. **Ningxin Zhao**: Data curation: Supporting; Writing – review & editing: Supporting. **Yang Lei**: Investigation: Supporting; Writing – review & editing: Supporting. **Xiujie Yang**: Data curation: Equal; Funding acquisition: Supporting; Supervision: Equal; Writing – review & editing: Supporting. **Xiangzhi Meng**: Data curation: Equal; Funding acquisition: Supporting. **Guosheng Ding**: Conceptualization: Supporting; Data curation: Equal; Funding acquisition: Supporting; Supervision: Supporting; Writing – review & editing: Supporting.

## Supplementary Material



## TECHNICAL TERMS

Language network: A set of brain regions in the frontal and temporal lobes that selectively respond to and process language.
Single-component coding: A neural coding scheme where specific brain regions are dedicated to processing one distinct linguistic function.
Combination-based coding: A neural coding scheme where individual brain regions simultaneously process and integrate multiple linguistic functions.
Functional clustering: A data-driven method that groups brain regions by similarity in their neural response profiles across conditions.
Hierarchical reading task: An experimental paradigm using stimuli of increasing linguistic complexity, from pseudowords to stories.
Situation model: A mental representation of the events, characters, and context described in a discourse or narrative.
Representational similarity analysis (RSA): A method comparing patterns of brain activity across conditions to reveal how the brain encodes information.
Syntactic processing: The cognitive process of analyzing grammatical rules and sentence structure during language comprehension.
SHAP (Shapley Additive Explanations): A statistical method that quantifies each variable’s individual contribution to a predictive model’s output.
Semantic processing: The cognitive process of extracting and representing word meaning during language comprehension.
