# Supplementary material for: Neural Coding of Multiple Linguistic Components During Reading Comprehension Across Development
Source: Neurobiol Lang (Camb). 2026 Sep 14;7:NOL.a.286. doi: 10.1162/NOL.a.286 (PMC13612845; doi:10.1162/NOL.a.286)
Supplement: Supplementary file 1 [file nol-07-286-s001.docx]

**Supplementary Materials**

In the adult group, the word > pseudoword contrast predominantly engaged the left middle temporal gyrus, the left precentral gyrus, the left inferior frontal gyrus (pars opercularis), the right middle temporal pole, and the right cerebellum. The sentence > pseudoword contrast activated the right middle temporal pole, the left middle temporal gyrus, and the right calcarine cortex, while the story > pseudoword contrast recruited a broader set of regions including bilateral middle temporal gyrus, the left inferior frontal gyrus, the left precentral gyrus, the left lingual gyrus, and the right cerebellum. Critically, the pairwise contrasts further isolated subcomponent-specific activations beyond those identified in the word > pseudoword contrast, with the sentence > word contrast selectively engaging the left middle temporal gyrus for syntactic processing and the story > sentence contrast recruiting the right insula and the left postcentral gyrus for situation model processing.

**Fig. S1.** Whole-brain activation maps in adults.

**(A–C)** Activation maps for each condition contrasted against the pseudoword baseline: (A) Word > Pseudoword, (B) Sentence > Pseudoword, (C) Story > Pseudoword. **(D–E)** Activation maps for between-condition contrasts: (D) Sentence > Word, (E) Story > Sentence. The Word > Pseudoword contrast was already presented in panel A and thus not repeated. All maps were displayed on a glass brain with the language network parcels outlined in gray (Fedorenko et al., 2010). Statistical maps were thresholded at voxel-level uncorrected p < .001 with cluster-level FWE-corrected p < .05. For the Sentence > Word contrast (D), no clusters survived the cluster-level FWE correction; therefore, results are shown at voxel-level uncorrected p < .001 with a minimum cluster extent of 30 voxels.

In the children group, the word > pseudoword contrast activated the left middle temporal gyrus, the right middle temporal pole, and the right superior temporal gyrus. The sentence > pseudoword contrast engaged the right middle temporal gyrus, the left superior temporal gyrus, and the bilateral cerebellum, while the story > pseudoword contrast recruited the bilateral middle temporal gyrus and the right cerebellum. The pairwise contrasts similarly isolated subcomponent-specific activations beyond those identified in the word > pseudoword contrast, with the sentence > word contrast activating bilateral middle temporal gyrus and the story > sentence contrast selectively engaging the temporal lobe.

**Fig. S2.** Whole-brain activation maps in children.

(A–C) Activation maps for each condition contrasted against the pseudoword baseline: (A) Word > Pseudoword, (B) Sentence > Pseudoword, (C) Story > Pseudoword. (D–E) Activation maps for between-condition contrasts: (D) Sentence > Word, (E) Story > Sentence. The Word > Pseudoword contrast was already presented in panel A and thus not repeated. All maps were displayed on a glass brain with the language network parcels outlined in gray (Fedorenko et al., 2010). Statistical maps were thresholded at voxel-level uncorrected *p* < .001 with cluster-level FWE-corrected *p* < .05. For the Story > Sentence contrast (E), no clusters survived the cluster-level FWE correction; therefore, results are shown at voxel-level uncorrected *p* < .001 with a minimum cluster extent of 30 voxels.

**Figure S3.** Cognitive Model Importance Across Three Functional Clusters. Horizontal bar plots illustrate the mean absolute SHAP values for each cognitive model and thus reflect their relative importance in predicting condition distance matrix within each functional cluster. Models are displayed in descending order of importance, and statistical significance is assessed through permutation testing with false discovery rate (FDR) correction, with significance levels indicated as *p* < .05, p < .01, and *p* < .001. Blue bars correspond to the children’s group whereas orange bars correspond to the adults’ group, and the clusters are defined as Cluster 1 comprising right frontal–temporal regions, Cluster 2 comprising left anterior frontal–temporal and right posterior temporal regions, and Cluster 3 comprising left anterior frontal–temporal and right posterior temporal regions.

**Figure S4.** Cognitive Model Importance Across Three Functional Clusters (Ridge Regression Results). Horizontal bar plots illustrate the mean absolute SHAP values for each cognitive model, reflecting their relative importance in predicting the condition distance matrix within each functional cluster. Models are displayed in descending order of importance, and statistical significance is assessed through permutation testing with false discovery rate (FDR) correction, with significance levels indicated as *p* < .05, *p* < .01, and *p* < .001. Blue bars correspond to the children’s group, whereas orange bars correspond to the adults’ group. The clusters are defined as Cluster 1 comprising right frontal–temporal regions, Cluster 2 comprising left anterior frontal–temporal and right posterior temporal regions, and Cluster 3 comprising left anterior frontal–temporal and right posterior temporal regions.

**Figure S5.** Cognitive Model Importance Across Three Functional Clusters (Random Forest Results). Horizontal bar plots illustrate the mean absolute SHAP values for each cognitive model, reflecting their relative importance in predicting the condition distance matrix within each functional cluster. Models are displayed in descending order of importance, and statistical significance is assessed through permutation testing with false discovery rate (FDR) correction, with significance levels indicated as *p* < .05, *p* < .01, and *p* < .001. Blue bars correspond to the children’s group, whereas orange bars correspond to the adults’ group. The clusters are defined as Cluster 1 comprising right frontal–temporal regions, Cluster 2 comprising left anterior frontal–temporal and right posterior temporal regions, and Cluster 3 comprising left anterior frontal–temporal and right posterior temporal regions.

**Figure S6.** Frequency distributions of behavioral measures for children (blue) and adults (orange). Histograms display the probability density distribution of each variable. Overlaid curves represent kernel density estimates. Dashed vertical lines indicate group means. RAN = Rapid Automatized Naming (seconds); CRF = Character Reading Fluency (number of characters correctly read per unit time); VF = Verbal Fluency (number of words produced).
